# Measuring the influence of contrast, ambiguity, and side of spatial context on perceptual dominance during binocular rivalry

**DOI:** 10.1167/jov.25.3.6

**Published:** 2025-03-21

**Authors:** Lisa Beckmann, Thomas Schenk, Karin Ludwig

**Affiliations:** 1 Clinical Neuropsychology, Department of Psychology, Ludwig-Maximilians-Universität München, Munich, Germany

**Keywords:** binocular rivalry, surround suppression, center-surround interaction, figure-ground segregation, bistable perception, pseudoneglect

## Abstract

The perception of ambiguous stimuli, notably binocular rivalry (BR), has been demonstrated to be influenced by spatial context. Previous results are, however, inconsistent with regard to whether the context biases perception toward the BR target that matches the context or toward the one that differs from the context. Furthermore, it is unclear what roles the perceptual ambiguity of the context and its lateral location play. We varied ambiguity, contrast, size, and location (left/right) of the context surrounding a rivalry target in a high-powered within-subject design to investigate (1) the effects of contrast and ambiguity of the surrounding context and (2) whether we could find laterality effects that correlated with an established pseudoneglect measure. The results showed an increase in perceptual predominance of the rivalry targets differing from the surround across all high-contrast conditions (irrespective of the ambiguity of the context), whereas the effect in the low-contrast condition was less pronounced. We found no laterality effects, but the strength of the context effects scaled with the context size (half/full). In an exploratory analysis, we further found a nasal advantage in the half-field condition. We interpret the results in the theoretical frameworks of surround inhibition and facilitation, figure-ground segregation, pseudoneglect, and nasal visual field advantage.

## Introduction

Humans navigate a challenging visual environment. In low light, unclear, or even normal conditions, perception needs to be constructed from sparse visual input. An example often used in experiments to uncover these constructive processes is perceptual ambiguity. Although the physical stimulus remains the same, perception fluctuates between possible interpretations, showing that perception is more than a mere reflection of input from the outside. This perceptual ambiguity can be created by different means. Famous visual phenomena are Rubin's vase (Rubin, 1915), an example of ambiguous figures (Andrews, Schluppeck, Homfray, Matthews, & Blakemore, 2002; Hasson, Hendler, Ben Bashat, & Malach, 2001), or structure-from-motion, sometimes also referred to as the kinetic depth effect (Andersen & Bradley, 1998). Another famous method is *binocular rivalry (BR)*. Here, one stimulus (e.g., an image of a house) is presented to one eye and another stimulus (e.g., an image of a face) to the other. As is the case for the other bistable phenomena, the subjective perception alternates between the two stimuli during their presentation (Brascamp, Klink, & Levelt, 2015; Levelt, 1965). Bistable perception offers a unique window into the dynamic interplay between sensory input and cognitive processes, providing theoretical insights into the mechanisms of perception and how the brain resolves conflicting sensory information.

### Spatial context influences perception

A general challenge of the visual system is that it receives information about the three-dimensional (3D) world around us, which is projected in two dimensions (2D) onto our retinae. Put differently, the visual system must identify 3D objects (distal stimulus) on the basis of 2D-light distribution on the retinae (proximal stimulus). The difficulty is that different distal stimuli can create the same proximal stimulus. The sensory stimulation alone is thus insufficient to establish the percept. Selection of a single percept from a variety of possible perceptual interpretations therefore requires the use of additional cues and constraints. This process of disambiguation can be best studied using bistable images. The different versions of bistable phenomena share the need for the stimulus to be disambiguated because two or more interpretations cannot be seen simultaneously. To help with this endeavor, further cues in the environment are recruited to determine which of two rivaling stimuli is more likely (e.g., Kim & Kim, 2019; Parise & Ernst, 2017). These cues range from low-level effects, such as lateral interactions between neighboring stimuli, to higher-level top-down effects that increase the salience of one of the rivaling interpretations (Tong, Meng, & Blake, 2006). These cues can even come from a different modality. Thus olfactory (Zhou, Jiang, He, & Chen, 2010), auditory (Einhäuser, Methfessel, & Bendixen, 2017), and tactile (Lunghi & Alais, 2013) cues influence the visual predominance patterns toward a congruent interpretation. However, the majority of research in this area examines within-modality influences of context, specifically the effect of visual context on visual perception. Such visual context effects can occur at different levels of visual processing. Visual-spatial cues are particularly relevant cues for the observer because they show the observer how different elements in a visual scene are related to one another regarding position, distance, and orientation. They provide important information about the structure and organization of a given scene, which can influence our percepts and actions.

In the case of two rivaling targets, two opposing spatial context effects have been reported: a matching boost effect (the target that is more similar to the context is favored and will become dominant, e.g., Grossmann & Dobbins, 2003) and a nonmatching boost effect (the target that is more different from the context is selected, e.g., Carter, Campbell, Liu, & Wallis, 2004). In the following paragraphs, an overview of the findings will be presented. Specific research articles will be highlighted to underline differences in resulting predominance biases because of presentation style, ambiguity of context, and contrast; for a more detailed overview, please refer to Table 1. Most notably, both matching and nonmatching boost effects could be found, each typically produced by a different type of paradigm.

**Table 1.** Overview of the current literature sorted by presentation, ambiguity, contrast, and resulting bias.

Presentation	Ambiguity	Contrast	Matching boost	Nonmatching boost
Side-by-Side	Nonambiguous	High	Alais and Blake (1998): Exp. 2^1^	Alais and Blake (1998): Exp. 1^1^
			Dieter et al. (2015): Exp. 1^1^	
			Sobel and Blake (2002): Exp. 1^1^	
		Low		
	Ambiguous	High	Alais and Blake (1999): Exp. 1-4^1^, Grossmann and Dobbins (2003): Exp. 1a-c^2^, 1d^3^, 2a^2^, 2b^2,3^, 2c^2^, 3-5^2^, 7-8^2^	
			Stuit et al. (2011): Exp. 1^1^, 2^1^	
			Whittle (1968): Exp. 3^1^	
		Low	Alais and Blake (1999): Exp. 2-4^1^	
Center-Surround	Nonambiguous	High	Baker and Graf (2010): Exp. 1-2^4^	Baker and Graf (2008): Exp. 1^1^
			Graf and Adams (2008): Exp. 1^1^, 2^2^	Carter et al. (2004): Exp. 1-3b^1^
				Dieter et al. (2015): Exp. 2^1^
				Fukuda and Blake (1992): Exp. 3^1^
				Hong and Shevell (2008): Exp. 1-2^1^
				Ichihara and Goryo (1978): Exp. 1-2^1^
				Mapperson and Lovegrove (1991): Exp. 1-2^1^
				Ooi and He (2006): Exp. 1-3^1^
				Paffen, Alais et al. (2005): Exp. 1^1^
				Paffen et al. (2004): Exp. 1^1^
				Paffen, van der Smagt et al. (2005): Exp. 1^1^
				Paffen et al. (2006): Exp. 1^1^, 3^1^
				Sereno and Sereno (1999): Exp. 1^2^
				Xu et al. (2010): Exp. 1-2^1^
		Low	Paffen et al. (2006): Exp. 1^1^, 3^1^	
	Ambiguous	High		
		Low		

*Note.* 1 = BR, 2 = structure-from-motion, 3 = ambiguous figures, 4 = bistable motion. Some studies show unclear/insignificant biases in certain conditions, e.g., Grossmann & Dobbins find significant effect of coupling between an ambiguous figure and a nonambiguous version (Exp. 1a, 1b, 3, 6) and Dieter et al. (2015) find that surrounding a BR stimulus with four gratings had no influence (Exp. 2).

When using side-by-side context configurations (e.g., Grossmann & Dobbins, 2003), matching boost effects dominate, whereas center-surround configurations (e.g., Carter et al., 2004) tend to favor nonmatching boost effects, although less consistently (e.g., Graf & Adams, 2008). The matching boost effect in studies using side-by-side configurations is quite robust, and it appears regardless of variations in the stimulus configuration and response mode (i.e., whether participants have to report coupling or the perceptual dominance of either target) (e.g., Alais & Blake, 1999; Grossmann & Dobbins, 2003).

In center-surround configurations, spatial context is usually provided as an annulus that surrounds the central ambiguous target (Paffen, Tadin, te Pas, Blake, & Verstraten, 2006). However, it can also consist of a surrounding grating that fills the entire screen (Carter et al., 2004). In the case of high-contrast static targets and surrounds, a bias for the targets not matched to the surround can be found (e.g., Carter et al., 2004; Fukuda & Blake, 1992; Ichihara & Goryo, 1978; Paffen, Alais, & Verstraten, 2005). However, there also seem to be some exceptions to this bias. For example, in the case of motion stimuli, the bias can be in the opposite direction (i.e., the motion that is most similar to the motion of the annulus is perceptually dominant) (Baker & Graf, 2010). The preference for the nonmatching target in the case of center-surround configurations in high-contrast conditions has been suggested to be due to figure-ground segregation. This is suspected to be due to lateral inhibition (of the matching grating) (Cavanaugh, Bair, & Anthony Movshon, 2002; Polat & Sagi, 1993), which suggests that at some level of visual processing, neurons that are strongly stimulated by a high-intensity background of the rivalry target suppress the less strongly stimulated neurons responding to the inner grating, the rivalry target. Alternatively, the figure-ground segregation could also be explained by results from single-unit neurophysiological experiments (Jones, Grieve, Wang, & Sillito, 2001; see Alais, 2012) showing that the response of an optimally driven cell is reduced when it is surrounded by a matched grating (Jones et al., 2001) whereas a surrounding of an orthogonal response can cause a supra-optimal response, leading to disinhibition or even facilitation (here: of the nonmatching grating) (Sillito, Grieve, Jones, Cudeiro, & Davls, 1995). These explanations are not mutually exclusive and could work in unison.

But, as mentioned above, the nonmatching boost effect does not always prevail in center-surround configurations. Evidence suggests that it is modified by contrast. The effect is especially strong when the contrast of center and surround is high. However, when center and surround are presented at a low-contrast, the effect seems to be reduced or even reversed. Paffen and colleagues investigated center-surround interactions in orientation, motion, and color in a BR paradigm (Paffen et al., 2006). The central rivalry targets and the surround were both either of high or low-contrast. When participants viewed the surround at high-contrast, target gratings matched to the surround were less dominant than a nonmatching rivalry target. However, when presenting the target and surround at low-contrast, this effect changed. How much it changed depended on the specific stimulus property. In the case of color, the nonmatching boost effect disappeared when low-contrast was used. In the case of orientation and motion, the effect was reversed: Reducing the contrast for these properties produced a matching boost effect (Paffen et al., 2006). These results suggest that in situations with lowered discriminability of the rivalry target or the surround, the surround effect can be weakened and even turned around into a facilitative effect. This could be due to the fact that low discriminability requires evidence to be collected over a wider retinal region to determine the percept (Sceniak, Ringach, Hawken, & Shapley, 1999). This facilitative effect could also occur when low discriminability is operationalized differently, such as through ambiguity (i.e., an ambiguous surround). In such cases, the unclear perceptual situation may necessitate the integration of information from both the center and the surround. Similar to low-contrast situations, a matching boost effect might thus prevail in case of an ambiguous surround. As seen by the empty rows in Table 1, the effect of surround ambiguity has not been investigated for center-surround configurations, with one inconclusive exception (Fukuda & Blake, 1992). Although their study used an ambiguous surround, the surround either completed none of the central stimuli or completed both central stimuli within the same condition. Thus it is impossible to draw conclusions concerning a nonmatching or matching boost effect from their ambiguous condition.

As discussed, spatial context can lead to either a matching boost bias or a nonmatching boost bias. Both of these effects can be due to one grating increasing in the average dominance duration, the other grating decreasing in dominance duration, or a combination of both. According to the revision of Levelt's second law (Brascamp et al., 2015; Levelt, 1965), this change results from an increase in the average dominance duration of the stronger stimulus (in our case, the more-facilitated or less-suppressed stimulus). In the case of a nonmatching boost bias, previous literature shows effects driven by an increase in the dominance duration of the nonmatching target or a combination of both an increase of the nonmatching and a decrease of the matching target (e.g., Carter et al., 2004; Ooi & He, 2006; Paffen, te Pas, Kanai, van der Smagt, & Verstraten, 2004; Paffen, van der Smagt, Pas, & Verstraten, 2005).

Although contextual influence can be based on different processes, it can also occur at different points of the visual processing stream. One way to investigate whether processing at lower or higher levels affected the predominance of a target is to observe differences between intraocular (relevant surround and center are in the same eye) or interocular (different eye) conditions. A stronger effect in the intraocular condition would implicate lower-level effects, whereas the presence of an interocular effect can be taken as an indicator of involvement of higher-level processes. Evidence for these effects comes from a binocular rivalry experiment, in which Paffen and colleagues found an increase in suppression depth (Paffen, Alais, et al., 2005): The suppression depth was the weakest in the interocular condition. It then grew stronger during the intraocular condition and was the strongest during the binocular (surround in both eyes) condition (Paffen, Alais, et al., 2005). Generally, the literature reporting ocularity effects agrees on a stronger intraocular and binocular center-surround interaction compared to interocular interactions (Carter et al., 2004; Dieter, Melnick, & Tadin, 2015; Grossmann & Dobbins, 2003; Sobel & Blake, 2002).

### Lateralized attention—pseudoneglect

Although substantial research has explored the contextual influence in bistable perception, little is known about the influence of the spatial position of said context (i.e., how the lateral position of the context stimulus modulates its influence). The potential relevance of the effect of the lateral position or more specifically, the effect of hemispace (i.e., right-side vs. left-side presentation) of the context is suggested by the occurrence of robust hemispace asymmetries in other perceptual tasks (e.g., the line bisection tasks, landmark tasks) (e.g., Benwell, Thut, Grant, & Harvey, 2014; Learmonth, Thut, Benwell, & Harvey, 2015), and lateralized visual detection tasks (e.g., Learmonth, Thut, et al., 2015). They are thought to be connected to spatial attention and examining them can provide valuable insights into the role of attentional processes in these tasks.

Research indicates visual field asymmetries, with healthy human observers favoring the left side or the lower half-field (Levine & Mcanany, 2005; Zito, Cazzoli, Müri, Mosimann, & Nef, 2016). These asymmetries in favor of the left side extend to various processes, including face processing (Hsiao & Cottrell, 2008; Prete et al., 2015) and spatial information (Heilman & van den Abell, 1980). Furthermore, a preference for the left can also be observed regarding contrast sensitivity (Rao, Rourke, & Whitman, 1981). Here, contrast thresholds are lower in the left half-field, compared to the right half-field with increasing temporal variation of the grating. These biases can also be found to extend to the attentional domain. For example, participants tend to start to explore visual displays on the left (Chiffi et al., 2021; Nuthmann, 2014). This attentional bias is called pseudoneglect based on classic findings in line bisection tasks (Bowers & Heilman, 1980). The term attributes leftward errors to an excessive attention to the left side, in analogy to clinical spatial neglect patients, who, after suffering from a right hemispheric brain injury, experience the employment of very little attention to the left and direct most of their attention to their right half-field (Bowers & Heilman, 1980; Jewell & McCourt, 2000). Pseudoneglect extends beyond the visual domain (Chiffi et al., 2021; Ludwig, Schmid, & Schenk, 2020), manifesting also in other sensory modalities (e.g., in the tactile modality) (Bowers & Heilman, 1980). Lateralization effects (left/right) remain largely unexplored in BR, but because BR has been shown to be affected by attention (Li, Rankin, Rinzel, Carrasco, & Heeger, 2017; Zhang, Jamison, Engel, He, & He, 2011), we want to test whether a BR task can also pick up a spatial attentional asymmetry. We will examine this by presenting the influencing surround in different hemifields (either right or left side).

### Present study

Different factors can contribute to how strongly a surround influences the perception of an ambiguous center. First, we investigated contrast and ambiguity by examining the difference in predominance across our three main conditions (high-contrast, low-contrast, and ambiguous surround) and compared the predominance of the nonmatching central target for each condition to its respective baseline. Based on previous results, we expect a strong nonmatching boost effect in high-contrast surround conditions. In the low-contrast condition, this bias is expected to be reduced or even reversed. Similarly, we expect a reduction or a reversal of this nonmatching boost bias in the ambiguous surround condition (if it functions like low-contrast, i.e., if the crucial factor is the lowered discriminability, which they share (H1.1)).

Next, we aimed to identify the driving factor behind the potential matching-boost biases (i.e., whether any bias would rely on the matching or nonmatching grating increasing in dominance duration, decreasing in dominance duration, or a combination of both). To this end, we compared the average dominance durations of the matching and nonmatching gratings in each surround condition against their respective baselines (H1.2).

Ambiguity also offers an opportunity to explore the role of ocularity. Typically, ocularity in center-surround configurations is studied using monocular constellations, where the rivalling central target is surrounded by a matching or a nonmatching surround in one eye while the other eye sees a neutral (e.g., gray or black) surround. If the effect is similar irrespective of whether the surround is shown to the same eye as the central stimulus with which interactions are assumed or to the other eye, higher-level visual processes (above monocular channels) are inferred (Kovacs, Papathomas, Yang, & Feher, 1996). If the effect is stronger intraocularly than interocularly, lower-level monocular processes are assumed to play a role (Logothetis, Leopold, & Sheinberg, 1996). In our experiment, one eye is presented with a matching surround, whereas the other eye is presented with a surround that matches neither central grating. Similar to the monocular surround experiments, this setup allows us to investigate whether the influence of the matching grating occurs at monocular or binocular processing levels. We tested this in our ambiguous condition and expected there to be both an interocular and intraocular effect, with the intraocular effect being stronger than the interocular effect (H1.3).

To examine whether pseudoneglect plays a role in center-surround interactions, we compared the strength of influence of a lateralized spatial context (left vs. right), expecting a stronger bias when the spatial context is on the left side of the central rivalry target (H2.1). To confirm whether the possible bias in the BR task reflects pseudoneglect, we correlated the bias with the bisection error from a classic line bisection task, expecting a positive correlation between the bisection error and a possible BR bias (H2.2).

Finally, as low-level summation effects are observable in the early visual cortex (Murray, Boyaci, & Kersten, 2006; Vinke & Ling, 2020), we wonder whether similar effects might be reflected in our BR task. Thus we compare the predominance difference when context is provided only in a half-field versus a full surround. We expect the full surround to exert a stronger influence than half-field surrounds (H3). By examining these factors in a single large sample, we aim to establish a reliable data basis for future models and contribute to better understanding the disambiguation process, which is central to perception.

## Methods

### Participants

A total of 46 participants (30 females and 16 males) took part in this experiment. Nine of those participants took part in the piloting phase. Participation was voluntary and compensation for their time was provided (€10 per hour or course credit). Ages ranged from 19 to 34 years, with an average age of 24.91 years (*SD* = 3.46 years). An a priori power analysis was conducted to estimate an appropriate sample size. Using the G*Power Software (version 3.1.9.7, Faul, Erdfelder, Lang, & Buchner, 2007), we computed for a repeated measures ANOVA (rmANOVA) with within factors (assuming a medium effect size) a sample size of *N* = 43. Anticipating the possibility of having to exclude participants, we increased the sample size to *N* = 46. We measured only right-handed participants without psychological or neurological disorders. All participants had normal or corrected to normal visual acuity and used their right hand during the experiments. All participants gave informed written consent before participation. The experiment adhered to the principles of the Declaration of Helsinki and was approved by the local ethics board of the Department of Psychology of the Ludwig-Maximilians-Universität München.

### Apparatus

Stimuli were generated with Matlab 2018a using the Psychtoolbox version 3.0.16 (The MathWorks Inc., 2018) on an HP EliteDesk 800 G3 TWR computer. To establish binocular rivalry, we used a double-mirror stereoscope. Participants saw part of the right half of the screen through two mirrors with their right eye and, similarly, part of the left half of the screen with the left eye. An AOC LCD monitor (27 inches, 100 Hz refresh rate), which was used to present the stimuli, was placed 56 cm from the participant's eyes in the BR experiment (calculated as the sum of the distances between the monitor and first mirror, outer and inner mirror, and inner mirror and eye, respectively). In the line bisection task, the monitor was at a distance of 62 cm.

### Stimuli

The complete stimulus configuration (comprising target and surround stimulus) presented to each eye measured 5.11° visual angle (va). The rivalry target had a diameter of 2.05° va, which is small enough to reduce mixed perception to a minimum. The patterns of the stimuli were sine-wave gratings at either 90% (high-contrast condition) or 10% (low-contrast condition) Michaelson contrast. The central gratings were rotated 45° clockwise (CW, i.e., to the right) or counter-clockwise (CCW, i.e., to the left). The rivalry target and spatial surround were separated by a small gap (0.10° va). This made it easy for the observer to distinguish between target and surround stimulus. The stimuli were surrounded by a circular black frame with blue circles, the so-called fusion-frame that is known to improve binocular fusion (Carmel, Arcaro, Kastner, & Hasson, 2010). The gratings had a spatial frequency of 3.5 cycles/degree va. The mean luminance of all gratings at the position on the screen was 100 cd/m^2^ at full contrast and 101 cd/m^2^ at low-contrast. Further information on ensuring luminance uniformity can be found in the supplement.

**Figure 1.**
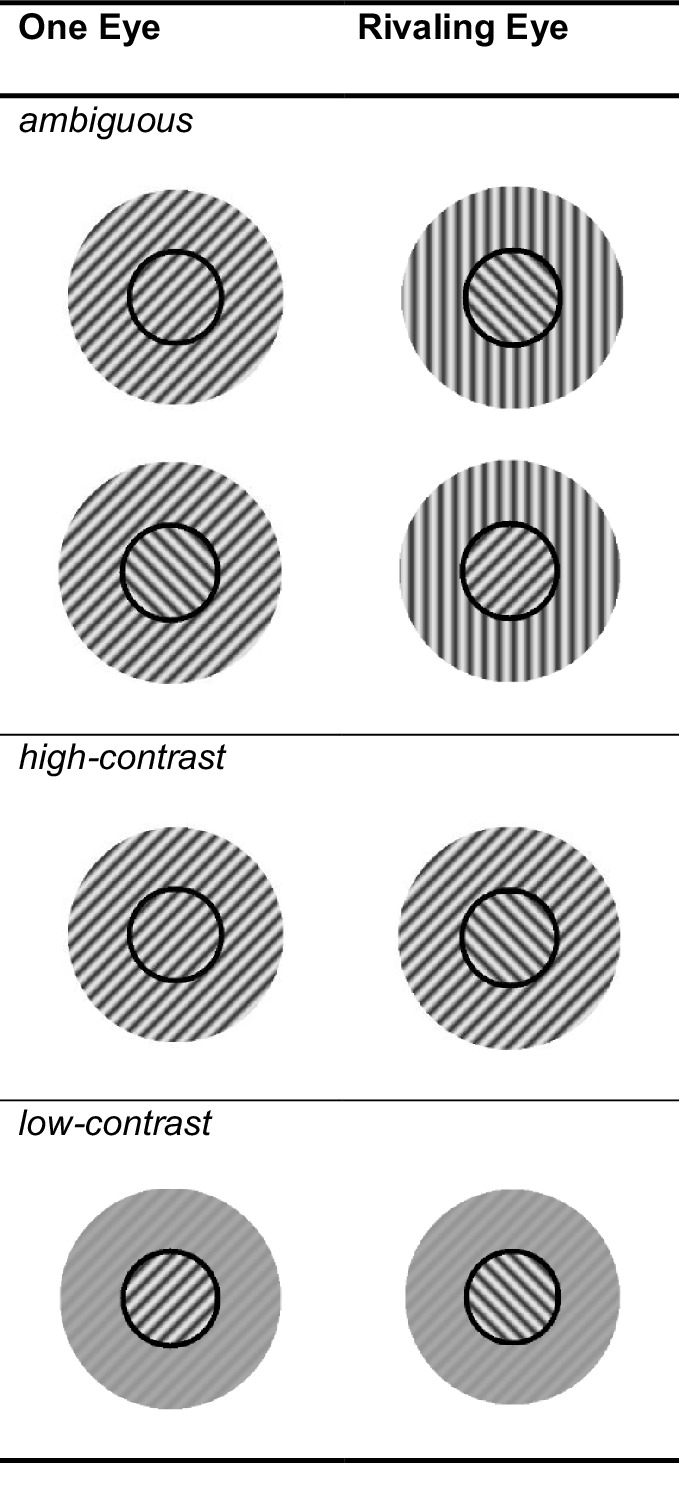
Example stimuli for the relevant conditions. Please note that this figure only shows a selection of the entire set of stimulus conditions. We varied to which eye which grating was shown and which surrounding grating matched one of the rivalry targets. For example, in the first row we could have also shown a CCW grating with a CCW surround on the right side. Additionally, these stimuli only show the full surround conditions. In the half-field condition, we only showed the surround to the left or right of the stimulus midline. A complete table of the full surround conditions is provided in the Supplemental Table S1 and for one of the half-field surrounds in the Supplemental Table S2.

In our experiments, we manipulated four different variables: (1) Surround Size: the surround could be full or partial (either only left or only right half-field). (2) Surround Ambiguity: the surround could be nonambiguous or ambiguous. (3) Surround Contrast: unambiguous stimuli had a high- or low-contrast surround. Examples of the high-contrast, low-contrast, and ambiguous conditions can be seen in Figure 1. In the ambiguous condition, we also varied (4) Ocularity: When the surround was ambiguous, only one grating was matched to one of the rivalry targets, while the other was a vertical grating. The matched surround could be presented in the same eye as the BR target with the same orientation (intraocular condition) or in the other eye (interocular condition).

**Figure 2.**
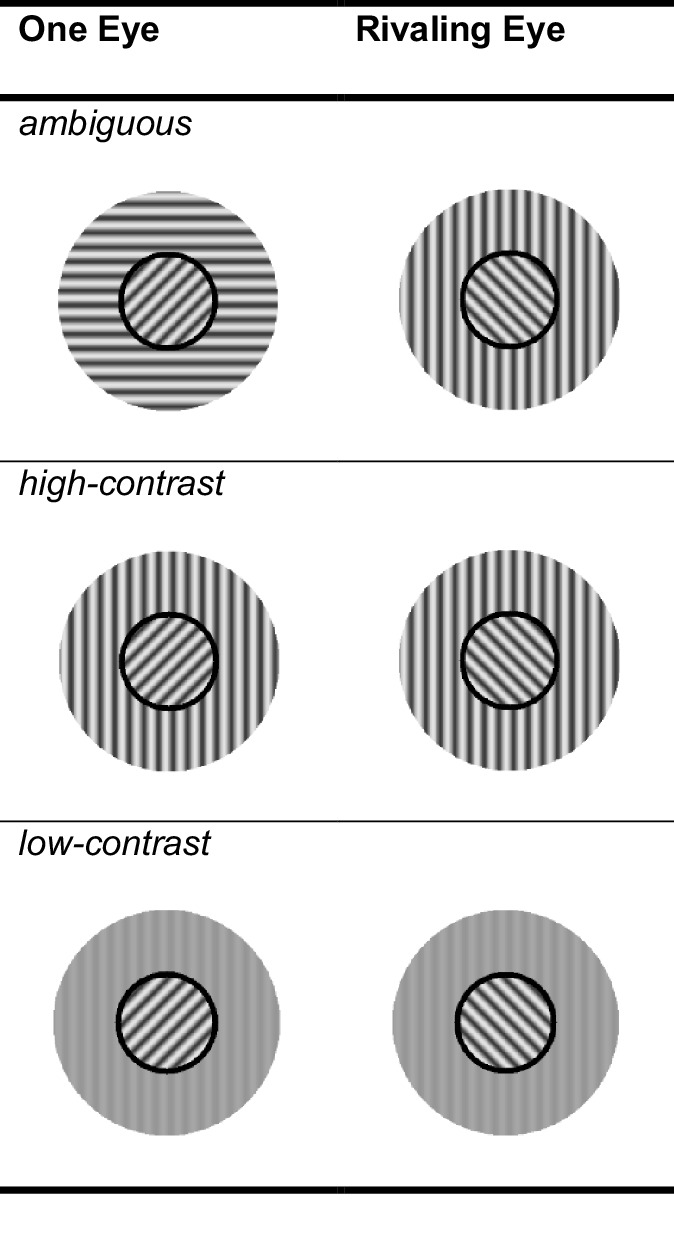
Example stimuli for the baseline conditions. Similar to Figure 1, only a selection of the entire set of stimulus conditions is presented here. We varied to which eye which grating was shown and which surrounding grating matched one of the rivalry targets. For example, we could have also shown a vertical surround around a CW target and a horizontal surround around a CCW target. Furthermore, these stimuli only show the full surround condition, in the half-field condition, we show the same gratings but only left or right of the rivalry target.

In addition to these stimuli with surrounds matched to one of the central gratings, we included baseline conditions with surrounds that matched neither of the central gratings. For each experimental condition, we designed a matching baseline-condition, see Figure 2. Accordingly, the baselines were ambiguous, high-contrast, or low-contrast. The surround in the nonambiguous baseline conditions was vertical instead of rotated, and, in the case of the ambiguous surround condition, one of the surrounds was vertical and the other horizontal. Additionally, we added a baseline condition that had no surround (NS) (i.e., just a black annulus). However, we did not need this condition in our current analyses, and it does not form part of the results.

### Procedures for the rivalry experiment

Participants took part in two sessions, each lasting roughly one hour. In the first session, participants participated in a computerized line bisection task and the first part of the BR experiment. In the second session, participants finished the BR experiment. The experiment was split into multiple sessions to avoid fatigue.

For the BR task, participants performed a practice trial without any surround before each session to familiarize themselves with the BR paradigm. Participants had the choice to repeat this trial as many times as needed. After the initial practice, the experimenter asked the participants whether they saw any alternations. In the case of participants negating this, the practice trial was repeated. Then, in each session, participants completed eight blocks with five trials per block lasting 50 seconds each. Participants were encouraged to take breaks between blocks. They completed a total of 80 trials (approximate duration: 66 minutes). All conditions were randomized across trials and blocks.

Participants were instructed to continuously report their perception of the circular rivalry target in the black circle presented at the center of the display. When participants saw the left-tilted grating, they were asked to press the left arrow key and keep it pressed for the length of their perception. Similarly, they were instructed to press the right arrow key for a right-tilted grating and the down arrow key for a mixed perception. All three keys were marked with glow-in-the-dark stickers. There were minor differences in the procedure between the first nine participants and the remaining sample described in the supplement.

### Procedures for the line bisection task

In the line bisection task (replication of Ludwig et al. (2020)), we used lines that can be categorized of four different lengths (i.e., short (8.67 cm long, 0.32 cm high), medium (13.03 cm long, 0.49 cm high), long (17.43 cm long, 0.65 cm high), and very long (21.86 cm long, 0.81 cm high)) presented at a distance of 62 cm shown in gray on a black background. All lines were vertically centered on the screen but varied in their horizontal position. The lines could be presented at the horizontal midline, 3.25 cm to the left or 3.25 cm to the right. Participants were instructed to perform the bisection by moving a mouse-controlled cursor, a triangle with a base and height of 1.5 cm. Participants were asked to click the mouse when the tip of the triangle was located at the point they perceived to be the midpoint of the line. The cursor started each trial at the bottom of the screen where, half the time, it was 3.25 cm to the left, and the other half, it was 3.25 cm to the right of the vertical midline of the computer monitor. Participants were instructed to be as accurate as possible and told there were no time restrictions. Clicking any mouse button finalized the bisection and initiated the subsequent trial, which started with an inter-trial interval (ITI) of 500 ms. Participants did not receive any feedback on bisection accuracy to avoid practice effects. After finishing the line bisection task, participants moved on to the BR experiment.

### Data analysis

As our primary dependent variable, we used the predominance of a given percept (e.g., matching/nonmatching to the surround, CW/CCW), which was calculated as follows (in the example for matched):

(1) $$\begin{array}{ccc} & & \;Predominance_{matching}\\ & & \;=\frac{total\;dominance_{matching}}{total\;dominance_{matching}+total\;dominance_{nonmatching}}\\ & & \;\; \end{array}$$

For some statistical comparisons, looking at the difference between the predominance of the matching and the nonmatching stimulus was more useful. This difference variable was computed in the following way:

(2) $$\begin{array}{ccc} & & \;\mathrm{Difference}\;in\;Predominance\\ & & \;=Predominance_{nonmatching}-Predominance_{matching}\;\; \end{array}$$

The individual values were averaged over all trials per relevant condition for each participant. To decide which measure of central tendency to use, we inspected the individual data distributions for each condition.

As another dependent variable, we used average dominance durations, calculated over all dominance durations (that were not artificially shortened by the end of the trial) in a given condition of interest across trials per participant. In conditions featuring matching and nonmatching contexts, we computed separate means for the two different types of contexts. For the baseline conditions, we averaged across all keypresses per condition. Even though dominance durations are typically not normally distributed, we chose to use the mean also for average dominance durations, as commonly done in the literature (e.g., Abuleil, McCulloch, Patterson, & Thompson, 2021; Brascamp, van Ee, Noest, Jacobs, & van den Berg, 2006; Metzger & Beck, 2020; Paffen, van der Smagt, et al., 2005; Qiu, Caldwell, You, & Mendola, 2020).

Whenever the prerequisites for parametric testing were violated according to a Shapiro-Wilk normality test, we performed a nonparametric alternative (Friedman test instead of repeated measures ANOVA (rmANOVA), Wilcoxon test instead of Student *t*-test, Kendall rank correlation instead of Pearson correlation). For rmANOVAs with more than one factor, no nonparametric alternative exists. However, it is recommended to use an aligned rank transformation before conducting the rmANOVA (Wobbrock, Findlater, Gergle, & Higgins, 2011). Additionally, when assumptions for parametric testing were met, we plotted the mean and standard error of the mean (as error bars) in the respective figures. When assumptions were violated, we used boxplots for the visualization.

### Data pre-processing

We excluded trials in which participants pressed no key for more than 30% of the time or reported more than 90% mixed predominance for a given trial. From the original 3720 trials, 3662 (98.44%) were left after applying these exclusion criteria.

## Results

### Context gives us cues about the most probable perception

First, we tested the most general assumption, namely that the three full context conditions would differ significantly in their bias from each other. For the high-contrast surround, we expected the central nonmatching grating to dominate perception, and for the low-contrast surround, we expected the surround-matched grating to be dominant and assumed an ambiguous surround would act similarly to a low-contrast surround (H1.1). Contrary to our predictions, the Friedman test did not reveal a significant effect of condition, χ^2^(2) = 3.95, *p* = 0.138, *W* = 0.04. However, post-hoc Wilcoxon tests did reveal a significantly bigger difference in predominance (nonmatching − matched percept) in the ambiguous condition compared to the low-contrast condition (*Z* = −1.79, *p*_adj_ = 0.037, *r* = 0.37). Because parts of our hypotheses were not met, we carried out two-sided tests from here on to be able to detect and describe effects that were opposite to those hypothesized.

**Figure 3.**
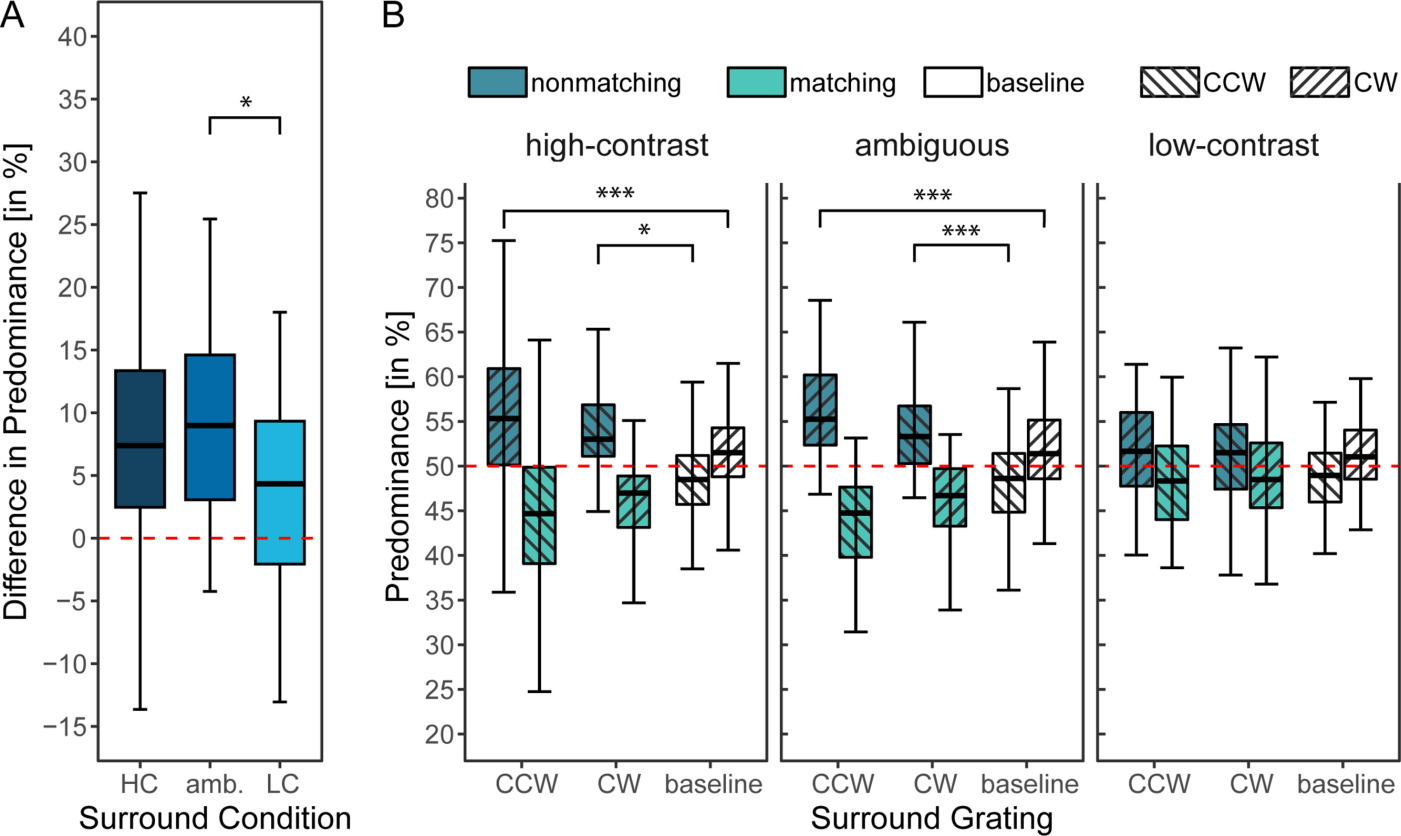
Predominance: Effect of full surround for different contrast and ambiguity conditions. Significance levels derived from Wilcoxon tests and adjusted via Bonferroni correction: *p < 0.05, **p < 0.01, ***p < 0.001. (**A**) The average difference in predominance (nonmatching – matching) of rivalry targets in different surround conditions. The x-axis represents the different surround conditions: high-contrast surround (HC), *ambiguous* surround (amb.), and low-contrast surround (LC). (**B**) Comparisons of the predominance of rivalry targets matching or nonmatching the surround separated per surround conditions and target orientation (CW, CCW). The predominance values of the nonmatching and the matching gratings always sum to 100%; the values of the matching gratings are only plotted for the sake of completeness. Thus tests were only carried out for one condition, i.e., nonmatching: high-contrast: CCW versus respective baseline: *Z* = −2.14, *p*_adj_ = 0.016, *r* = 0.35; CW versus respective baseline: *Z* = −3.18, *p*_adj_ < 0.001, *r* = 0.48; *ambiguous*: CCW versus respective baseline: *Z* = −3.09, *p*_adj_ < 0.001, *r* = 0.46; CW versus respective baseline: *Z* = −5.01, *p*_adj_ < 0.001, *r* = 0.70; low-contrast: CCW versus respective baseline: *Z* = −0.03, *p*_adj_ = 0.488, *r* = 0.10; CW versus respective baseline: *Z* = −1.48, *p*_adj_ = 0.070, *r* = 0.27. For clarity, outliers are not depicted. Individual participant data is available in Supplemental Figure S1.

As can be seen in Figure 3A, it was always the nonmatching grating that had greater perceptual predominance than the matching grating irrespective of condition. We tested whether this effect differed from chance level (50% predominance or 0% difference in predominance) for all three conditions. The Wilcoxon signed-rank tests revealed this to be the case in all conditions (high-contrast surround condition: *Z* = −3.55, *p* < 0.001, *r* = 0.53, ambiguous surround condition: *Z* = −5.20, *p* < 0.001, *r* = 0.72, low-contrast surround condition: *Z* = −2.34, *p* = 0.010, *r* = 0.38.

These findings were further corroborated by comparing all surround conditions to the respective baseline (two-sided tests) by comparing the predominance of the nonmatching stimulus to the predominance of the same grating (CW, CCW) in the baseline condition; see Figure 3B. For example, in the high-contrast condition, the nonmatching grating in 50% of the trials was a clockwise grating (CW). The predominance values in this condition were compared to the predominance values of the CW gratings in the baseline condition (i.e., the condition with a vertical surrounding grating [meaning that neither CW nor CCW gratings matched the surround]). In all comparisons, the condition of interest was significantly different from the baseline except for the low-contrast surround after Bonferroni correction for multiple testing, see Figure 3B and its caption.

**Figure 4.**
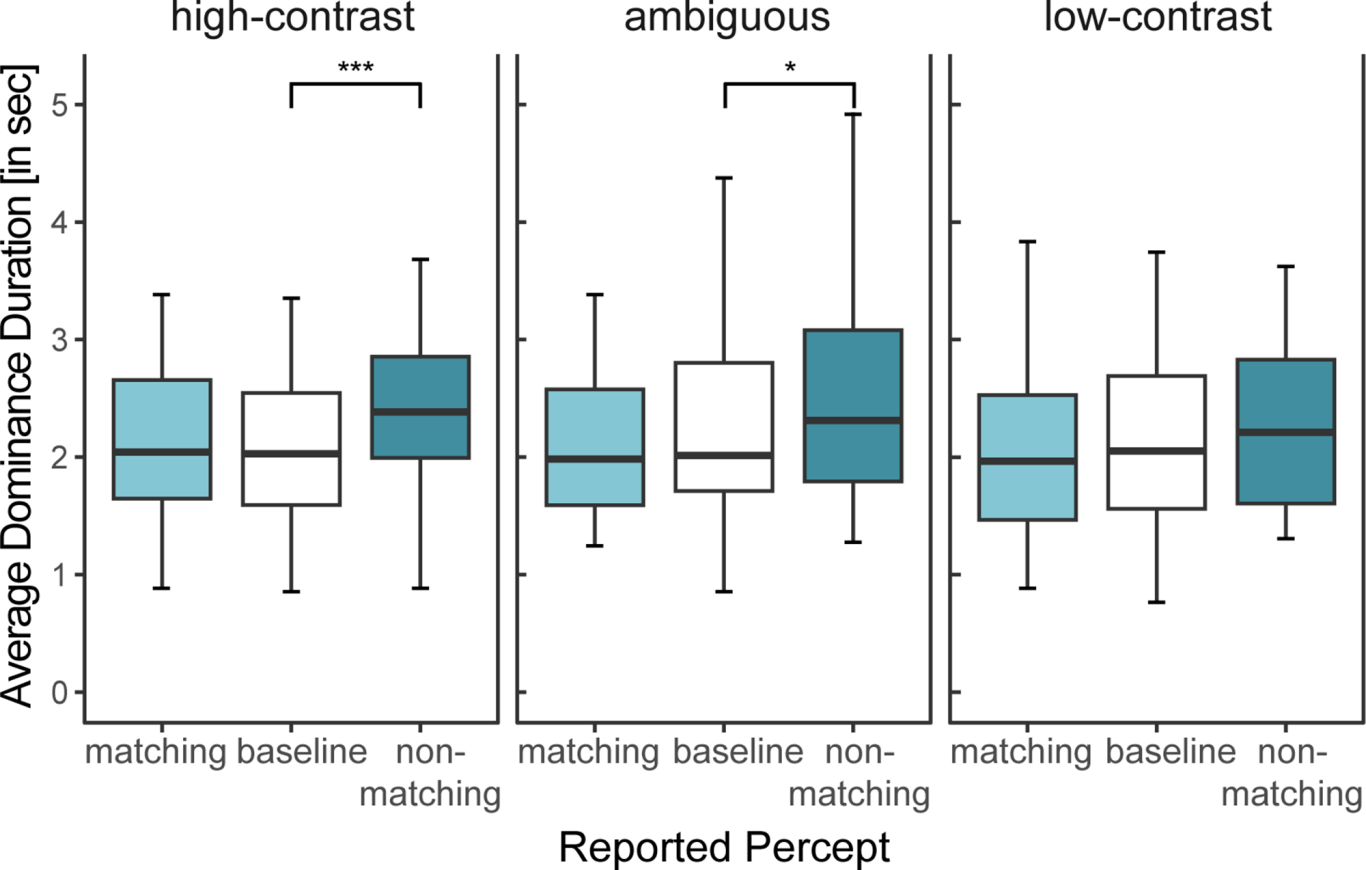
Mean dominance duration: Effect of full surround for different contrast and ambiguity conditions. Significance levels derived from Wilcoxon tests and adjusted via Bonferroni correction: *p < 0.05, **p < 0.01, ***p < 0.001. The x-axis represents the reported percept in a given surround condition. Results are pooled across different possible percepts (CW, CCW). Outliers are not depicted for clarity. Individual participant data are available in Supplemental Figure S2.

We can thus conclude that, in all conditions, the nonmatching grating was more dominant than the surround-matched grating and that for the two high-contrast surround conditions (nonambiguous and ambiguous), this difference was statistically significant. This effect could be due to two processes: either the dominance durations of the nonmatching grating became longer compared to the baseline, or the dominance durations of the matching grating became shorter (or a combination of the two). To determine which of the two effects was more prominent, we compared the average dominance durations of the conditions of interest to their respective baseline (H1.2). The results are summed up in Figure 4: the average dominance period of the nonmatching grating was significantly higher compared to the baseline, both for the high-contrast surround (*Z* = −3.94, *p*_adj_ < 0.001, *r* = 0.57) and the ambiguous surround (*Z* = −2.28, *p*_adj_ = 0.011, *r* = 0.34). None of the surround matching comparisons with the baseline became significant, nor did the comparison for the nonmatching grating in the low-contrast condition. Thus the perceptual predominance of the nonmatching grating can be traced back to longer mean dominance periods of this grating, at least in the high-contrast conditions (i.e., the high-contrast and the ambiguous condition).

In the ambiguous condition, the surround that matched one of the central gratings could be presented to the same eye as its central grating (intraocular) or to the other eye (interocular), see Figure 1. Whether the intraocular influence was stronger than the interocular influence or similar in strength can help us identify at what level of the visual processing stream the effect arises. We thus compared the mean effect of the grating matched to the surround (i.e., the difference between matched and unmatched) between intraocular (median difference = 7.41, inter quartile range (IQR) = 12.19) and interocular (median difference = 7.50, IQR = 12.16) conditions (H1.3). We found no significant difference (*Z* = −0.81, *p* = 0.415, *r* = 0.12). To further test whether this nonsignificant finding amounted to evidence for the null hypothesis (i.e., that intraocular and interocular influence were indistinguishable), we carried out a paired Bayesian Wilcoxon test. The calculated Bayes factor BF_01_ = 1.75. can be interpreted as weak or anecdotal evidence in favor of the null hypothesis (Jeffreys, 1998; Lee & Wagenmakers, 2013).

### Spatial context in different half-fields

In addition to the full surround condition, we measured conditions where the surround was only present on one side of the central grating (left or right). Assuming that healthy young participants show a slight attentional bias toward the left (pseudoneglect, Friedrich, Hunter, & Elias, 2018; Jewell & McCourt, 2000; Ludwig & Schenk, 2022), we hypothesized that the spatial surround on the left influenced the perception of the rivaling gratings in the center more strongly than the surround on the right (H2.1).

To test this statistically, we calculated a measure of the surround effect (difference between matching and nonmatching predominance). Given that our results above demonstrated a prolonged predominance of the nonmatching grating, we expected positive values. We predicted the surround effect value to be larger for trials with spatial context on the left than those with context on the right and calculated an rmANOVA with the factors surround type (ambiguous, high-contrast, low-contrast) and side of surround (left, right) on the aligned rank transformed data. Neither the factor surround type (*F*(2, 224.23) = 1.11, *p* = 0.332), nor the side-factor (*F*(1, 224.24) = 0.03, *p* = 0.859), nor the interaction (*F*(2, 224.23) = 0.74, *p* = 0.477) was significant.

**Figure 5.**
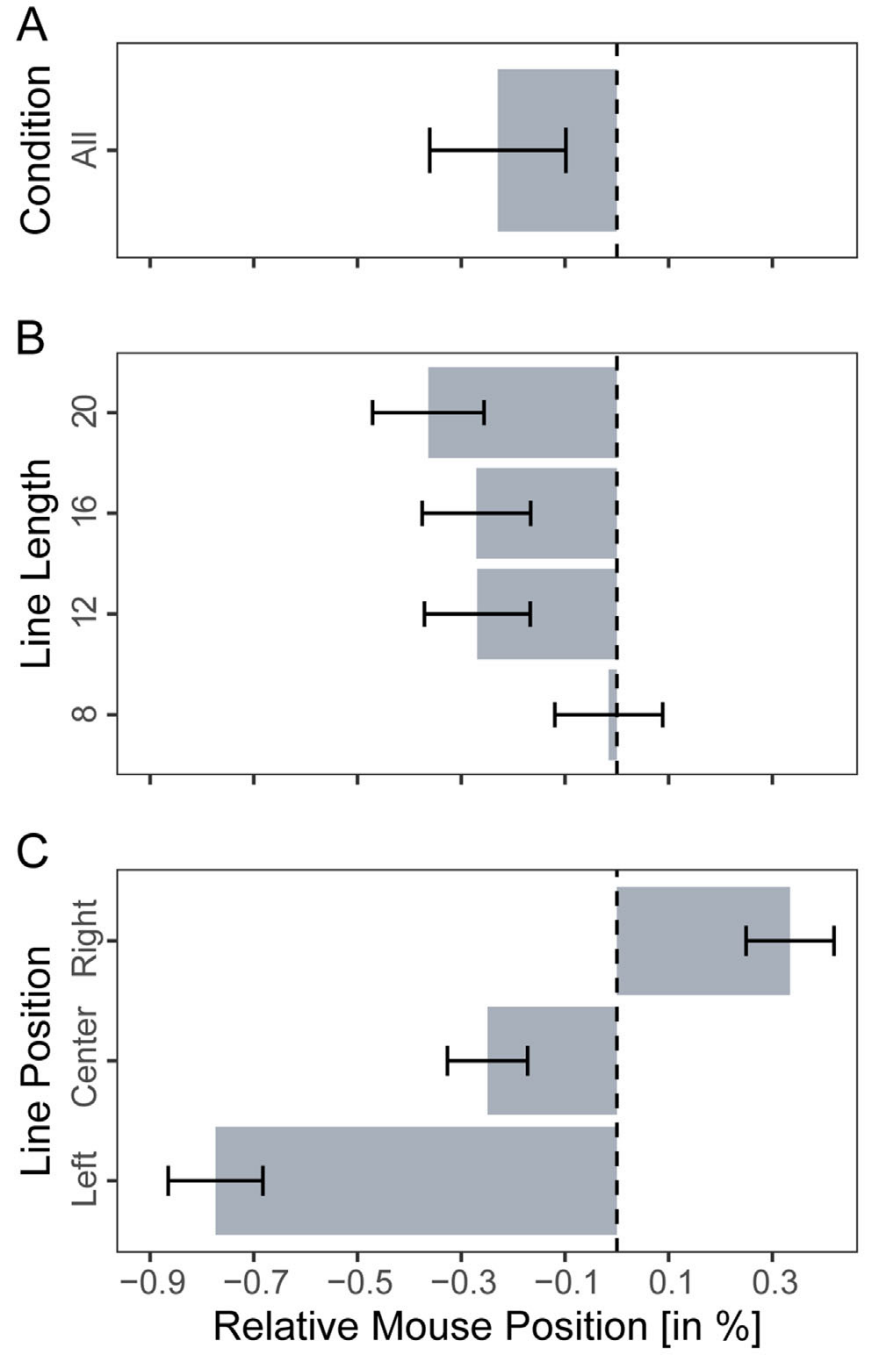
Results from the line bisection task. (**A**) Bisection errors in relative mouse position percent, over all trials, (**B**) split for line length, and (**C**) line position.

Although we did not find any effects of the half-field side on the group level, our participants showed some variation in the difference between the left and right surround effects. Thus we tested whether this difference correlated with a standard measure of pseudoneglect, namely line bisection errors (H2.2). Before correlating the side-dependent surround effects with this measure, we tested whether our participants showed a typical pattern of results (i.e., pseudoneglect and its modulation by features of the to-be-bisected line in this task). The experimental paradigm and the analysis were conducted in accordance with Ludwig and colleagues, and the results were very similar (Ludwig et al., 2020): The overall mean perceived midpoint of the line (bisection error [BE]) was −0.23% (the left endpoint of the line was labeled as −50% and the right as 50%), which differed significantly from zero, *t*(45) = −1.75, *p* = 0.043, Cohen's *d* = 0.26, see Figure 5A, thus demonstrating pseudoneglect. Furthermore, rmANOVA with Greenhouse-Geisser correction yielded a significant main effect of line length (*F*(2.01, 90.49) = 3.76, *p* = 0.027, *η*^2^ = 0.01, Figure 5B) and of line position (*F*(1.23, 55.27) = 70.80, *p* < 0.001, *η*^2^ = 0.14, Figure 5C), replicating previous results (Jewell & McCourt, 2000; Ludwig et al., 2020).

In the next step, we correlated the difference in the surround effect (left half-field – right half-field) with the overall BE averaged across all conditions and the BE of the lines where the strongest error was to be expected (i.e., BE of the longest lines and the leftmost lines). We calculated the Kendall tau rank correlation coefficient, but none of the values correlated significantly with the difference in the surround effect on all lines: *τ* = −0.01, *p* = 0.882, longest lines: *τ* = −0.04, *p* = 0.503, leftmost lines: *τ* = −0.04, *p* = 0.468), see Figures 5B and 5C.

McIntosh, Schindler, Birchall, and Milner (2005) proposed using their novel method for administering and analyzing line bisection, which gives an endpoint weighting bias (EWB). This can be used as a measure of attentional bias and has been shown to correlate significantly more highly with cancellation, copying, and drawing measures than the classical line bisection error and is thought to be a more precise measure of neglect (EWB > 0) and pseudoneglect (EWB < 0), (McIntosh, Ietswaart, & Milner, 2017). We calculated the EWB for each participant to further demonstrate their pseudoneglect, expecting an EWB smaller than 0, which could be confirmed with an average EWB of −0.012: *t*(45) = −2.59, *p* = 0.006, Cohen's *d* = 0.38. However, the EWB also did not correlate significantly with the difference in the surround effect (left half-field – right half-field), *τ* = −0.01, *p* = 0.940. To summarize, the left and right half-fields did not affect BR differently, and their effects did not correlate with classical pseudoneglect measures.

In an early study, Kaushall (1975) suggested a connection between the hemisphere mediating the faster switch rate and stimulus strength. In light of pseudoneglect, this means that the stimuli on the left should be processed more strongly, thus leading to a higher switch rate when the surround was presented to the left. This implies that the switch rate might be the more appropriate measure for detecting hemisphere differences. Therefore, to ensure we did not overlook any laterality effects because of selecting the wrong dependent variable, we conducted an exploratory analysis testing if there was a higher switch rate for the left half-field (mean = 17.79, *SD* = 7.03) compared to the right half-field (mean = 17.37, *SD* = 7.22) in the high-contrast surround conditions. The test only approached significance: *t*(45) = 1.64, *p* = 0.054, Cohen's *d* = 0.24.

### Comparing a full surround to a half-field surround

Our design further let us answer the additional question of how strong the influence of a half-field surround was compared to a full surround. We hypothesized that it would be stronger in the full surround condition compared to the half-field conditions (H3). As shown above, no significant differences exist between the left and right half-fields. We thus collapsed left and right half-field data to just a single factor of half-field.

**Figure 6.**
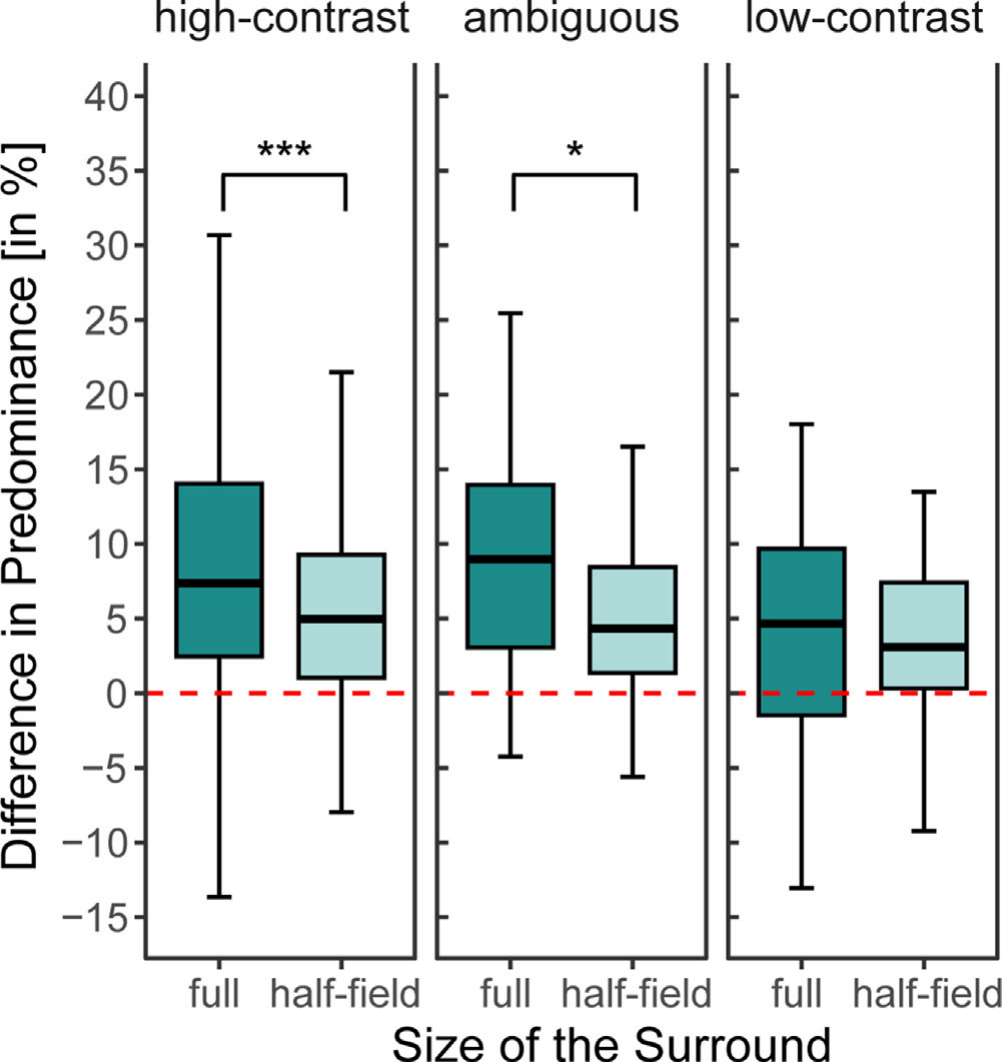
Results from the comparison between half- and full-field surrounds. Significance levels as derived from the Wilcoxon tests and corrected for by Bonferroni adjustments: *p < 0.05, **p < 0.01, ***p < 0.001. The mean difference in predominance is split by surround condition, comparing half-field and full surround. Individual participant data are available in Supplemental Figure S3.

We compared the full surround to the half-fields using three additional Wilcoxon tests. These tests revealed a significant effect in the high-contrast condition (half (Mdn = 4.15) vs. full (Mdn = 7.38): *Z* = −3.34, *p*_adj_ < 0.001, *r* = 0.48) and in the ambiguous condition (half (Mdn = 4.33) vs. full (Mdn = 8.98): *Z* = −1.89, *p*_adj_ = 0.029, *r* = 0.28). The low-contrast condition did not demonstrate a significant difference (half (Mdn = 3.09) vs. full (Mdn = 4.34): *Z* = −0.41, *p*_adj_ = 0.340, *r* = 0.06). This demonstrates a surround effect that is numerically approximately half for the half-field surround condition compared to the full surround condition (see Figure 6).

In an exploratory analysis, we tested a further effect: recently, Sahakian, Paffen, Van der Stigchel, and Gayet (2022) found a nasal visual field advantage in interocular competition in a breaking continuous flash suppression experiment. They found that targets presented in the nasal visual half-field broke through suppression faster than targets presented to the temporal visual half-field. We therefore tested whether a nasal advantage in the form of a stronger influence of the nasal surround on BR could be found for our data. For our stimulus configurations in the ambiguous condition, we define a stimulus as nasal when the grating that completes one of the central stimuli (i.e., CW or CCW grating) is presented in the nasal half-field of one eye. In this case, a vertical grating would be presented to the temporal half-field of the other eye. Conversely, a stimulus would be considered temporal when the CW or CCW grating was presented to the temporal half-field of one eye. Again, a vertical grating would then be presented in the nasal half-field of the other eye. To give a specific example, if the left eye perceives a central CW target with a left half-field surround that is made of a CCW grating, while the right eye sees a central CCW grating target with a left half-field surround that is a vertical grating, we classify this condition as temporal because of the CCW grating being presented in the temporal half-field in the left eye. In contrast, if the left eye sees a central CW target with a right half-field surround that is CCW, while the right eye sees a central CCW target with a right half-field surround that is a vertical grating, we would classify this as *nasal*, since the orthogonal grating is presented in the nasal half-field of the left eye. We give an overview of the (left) half-field stimuli with reference to the nasal/temporal condition in the Supplemental Table S2; the right half-field works analogously.

A Wilcoxon signed rank test confirmed the expected nasal preference: the difference between the matching and the nonmatching grating was stronger in the nasal condition (Mdn = 4.97) compared to the temporal condition (Mdn = 2.79): *Z* = −2.50, *p* = 0.006, *r* = 0.16. The nasal surround thus exerted a stronger influence on the central BR stimulus than the temporal surround*.* This finding confirms the nasal visual field advantage observed by Sahakian et al. (2022) and demonstrates that this nasal advantage for disambiguating cues is not restricted to the flash-suppression paradigm.

## Discussion

This study investigated the influence of spatial context on BR perception. The effects of contrast, ambiguity, size, and lateral location of the context were examined in a within-subject design. The results showed that the perceptual predominance of the nonmatching rivalry target increased in high-contrast conditions, irrespective of the ambiguity of the context. No laterality effects were found, but the context effects scaled with the size of the context. In addition, a nasal visual field advantage was observed in the half-field condition. These findings contribute to a better understanding of spatial context in ambiguous perception.

### Ambiguity does not act similarly to low-contrast

In the first part, we found that all surround conditions showed a predominance of the nonmatching grating that was above chance. This finding indicates that the surround interactions per se worked as expected for the high-contrast condition. In the low-contrast condition, we found a reduced effect of surround interactions while the preference for the nonmatching grating prevailed (H1.1). Previous studies have found a reversal of the effect when the contrast was low (Paffen, Tadin, et al., 2006). Differences in design could explain these slightly divergent results. Paffen and colleagues used a contrast much lower than ours in their low-contrast condition, 2% Michaelson contrast, compared to our 10% Michaelson surround contrast. This difference in contrast strength might explain why the authors found a switch to a matching boost effect, whereas our results only show a reduction of the nonmatching boost effect. Furthermore, Paffen and colleagues lowered the contrast of the rivalry target along with the contrast of the surround. In our design, we decided against lowering the rivalry target contrast for multiple reasons and kept it equally high between all conditions. It was our aim to compare the different surround conditions directly with each other and to assess thereby the specific contributions of differences in those surround stimuli on the perception of the target stimulus. It was, therefore, essential to avoid confounding changes in the surround with changes in the central target. It is, in fact, well-known that changes in contrast to the central target in itself can affect rivalry in several ways. Specifically, lowering the contrast of the target stimulus leads to, e.g., increased patchwork perception (Hollins, 1980; Skerswetat, Formankiewicz, & Waugh, 2016) and reduced switch rate (Alais, 2012; Breese, 1899; Levelt, 1965; Pettigrew, 2001; Skerswetat et al., 2016). We encountered the same during piloting. Additionally, we perceived phases in which the gratings faded. This aligns with the findings from Liu, Tyler, and Schor (1992), where the authors noted that rivalry perception was contingent on surpassing a certain contrast threshold (approximately 10% contrast at our spatial frequency). Below this threshold, participants reported perceiving a stable plaid, and at significantly lower contrasts, the stimuli were rendered imperceptible (Liu et al., 1992). Taken together these observations reinforce the rationale behind prioritizing high-contrast targets to achieve more reliable rivalry induction and to make the dependent variables more comparable between experimental conditions. However, we acknowledge that this profound change in design comes with disadvantages that might perhaps explain why we observed only a reduction in the nonmatching boost effect in the low-contrast condition but no effect reversal (i.e., a matching boost effect). Presenting a high-contrast center in front of a low-contrast surround might have increased the perceived difference between the surround and the target, thereby facilitating the segregation of the figure (central target) from the ground (surround).

In addition to the results by Paffen and colleagues (2006) and our results, further evidence for enhancement effects from low-contrast surround comes from contrast matching paradigms. In these paradigms, participants determine the contrast required for an isolated patch to equalize the apparent contrast of a central patch embedded in a different surround (e.g., Cannon & Fullenkamp, 1993; Snowden & Hammett, 1998; Xing & Heeger, 2001). Generally, a surround can either suppress or enhance the perceived contrast of a central patch (Xing & Heeger, 2001). These authors found that surround contrasts can lead to suppression, resulting in an increased contrast threshold for the central patch. This suppression effect depends on the relative orientations and spatial frequencies of the surround and the central patch (Chubb, Sperling, & Solomon, 1989). However, enhancement effects, which increase the apparent contrast of the central patch, do not depend on relative orientation (Xing & Heeger, 2001). This indicates that the interaction between the central patch and the surround can vary based on the specific visual contrast context, explaining why we could not find the reversal previously demonstrated by Paffen and colleagues (2006).

The strong predominance of the nonmatching grating can be due to two different processes. Either the average dominance duration of the nonmatching grating increases or the average dominance duration of the matching grating decreases. In our case, the average dominance duration of the nonmatching grating increased (H1.2). Generally, this is in line with the revision of Levelt's second law, which states: “Increasing the difference in stimulus strength between the two eyes will primarily act to increase the average perceptual dominance duration of the stronger stimulus” (Brascamp et al., 2015, p. 27). In our study, said modulation of “stimulus strength” was achieved by introducing the spatial surround, which strengthened the nonmatching grating and weakened the matching grating (through facilitation and inhibition processes, see below). Hence, our results perfectly confirm Levelt's second law (revised): the “stronger” stimulus (the nonmatching and thus less suppressed grating) was, on average, dominant for longer periods. This is in line with results from Paffen, van der Smagt, et al. (2005), who used motion stimuli that rivaled with static stimuli. The former could be suppressed by a same direction surround (if the surround was wide enough). This suppression led to an increase in the mean dominance durations of the rivaling stimulus (i.e., the static grating).

Different physiological effects can be at work to lead to this increase in dominance: Lateral inhibition could have weakened the matching grating, as it has been shown that the response of an optimally oriented stimulus presented to a neuron's classical receptive field in the primary visual cortex can be inhibited by presenting a surrounding annulus with a matched orientation (Blakemore & Tobin, 1972; Knierim & Van Essen, 1992). On the other hand, Sillito and colleagues (1995) made a significant observation in the macaque visual cortex. They found that when the surrounding grating was oriented orthogonally to central grating, not only was there an absence of surround suppression, but they also observed a facilitation effect. This facilitation resulted in neuronal responses that exceeded those elicited by the optimal stimulus for the classical receptive field alone. This finding suggests that the visual system may enhance the salience of orientation contrasts between the center and the surround. In the context of our nonmatching boosting effect, a similar facilitation effect could potentially strengthen the neural response to the nonmatching grating. This enhancement of nonmatching stimuli may serve to accentuate boundaries or edges in visual scenes. The interplay with lateral inhibition (which typically suppresses response to similar orientations in the surround) could contribute to the perceived difference in the strength of the stimuli. These mechanisms may play a crucial role in figure-ground segregation and the detection of orientation discontinuities in natural scenes (Sillito et al., 1995).

The outcomes observed between ambiguous surround and low-contrast surround conditions in center-surround interactions exhibit clear differences. Introducing an ambiguous surround did not lead to a reduced effect in the nonmatching boost effect that could be observed in the low-contrast condition. These disparities suggest that alternations in the nonmatching effect may be confined to luminance contrast rather than stemming from diminished perceptual saliency or discriminability. However, it is imperative to approach this conclusion with caution, as manipulating the ambiguity of the surround entails intricate interplays between the surround and the central rivalry target. Further explorations are warranted to gain insights into the underlying mechanisms driving the outcomes observed in the ambiguous condition.

The presence of an effect when the surround is presented interocularly, which we found suggests an involvement of higher cortical areas and can be interpreted as the surround effects going beyond low-level effects (H1.3). Not only is the effect present, but it even seems approximately equally strong as in the condition with the nonambiguous surround. This finding is surprising since this means that, in fact, it does not matter whether the surround grating matched to one of the rivalry targets is presented to the same eye as the rivalry target it is matched to or to the other. It always led to a relative increase in dominance of the nonmatching grating. Further evidence for higher-level processing comes from physiological studies, which showed that activity of binocular-oriented but not monocular-oriented cells correlated with perceptual alternations as the level of processing gets higher: 18% of binocular-oriented cells in V1/V2, 38% in V4, 43% in MT (Leopold & Logothetis, 1996), and 90% in STS and IT (Sheinberg & Logothetis, 1997). In a psychophysical experiment, Bonneh, Sagi, and Karni (2001) showed that for stimuli that were rapidly swapped between the eyes, pattern rivalry replaces eye rivalry as pattern coherence is increased. Taken together with the physiological results, this shows that the level of visual processing where competition arises and is consequently resolved is dictated by the degree of stimulus coherence (Bonneh et al., 2001) and moves up in the visual processing hierarchy. These results are in line with our strong interocular effect.

### BR in surround interactions is not a suitable measure of pseudoneglect

As lateralized biases can be found in various domains and offer a good idea on paradigms suitable for clinical unilateral neglect studies, we investigated whether we find a bias for the left in healthy participants when using spatial context in BR. To verify any potential bias in BR, we had participants complete a line bisection task. We directly replicated results from Ludwig and colleagues’ previous study, which included a line bisection task (Ludwig et al., 2020). However, we could not find a difference in the influence of context between the left and right half-field in the BR task (H2.1). Consequently, we found no correlations between the BR difference and the line bisection task (H2.2).

We are not the only group unable to demonstrate a bias in the left and right half-field in BR. In an early study, Kaushall (1975) used a BR stimulus with one line to the left and one to the right of fixation. These lines could be rotated CW or CCW and rivaled with the opposite orientation in the other eye (Kaushall, 1975). He did not find a bias in dominance weights for one hemisphere. However, he suggests that the hemisphere mediating the faster switch rate correlates with stimulus strength. With this measure, he did find a bias, indicating a stronger left hemisphere influence on full-field rivalry. One can assume that this is due to the stimuli on the left (in our case, the left surround) being processed more strongly and thus having a higher effect on the central target than stimuli on the right (in our case, the right surround). We took this as a suggestion to determine whether the alternation rate would indicate a preference for the left or right hemisphere. Our results went in the same direction but only approached significance.

In a recent review by Mitchell and colleagues, the inconsistent nature of pseudoneglect within individuals is highlighted, particularly across sensory modalities and tasks (Mitchell, Harris, Benstock, & Ales, 2020). Furthermore, Learmonth, Gallagher, Gibson, Thut, and Harvey (2015) could not find a reliable effect of pseudoneglect across different visual pseudoneglect tasks. They emphasize that pseudoneglect, as well as clinical unilateral neglect, is a multicomponent issue. Individuals who overestimate the size of the left hemispace as measured by line bisection or landmark tasks do not necessarily exhibit a substantial leftward overestimation of luminance or spatial frequency on the left. Neither do they show an increase in stimulus discrimination to this site. The variation in this bias between tasks may reflect a more complex interaction between dorsal and ventral attentional networks mediated by task demand variations (Learmonth, Gallagher, et al., 2015).

### Surround interactions scale with amount of visual input

Since we tested the spatial context as complete and half-field surrounds, the two conditions can be compared. Notably, the half-field surround produced an effect that was approximately half as strong as a full surround (*H3*). Similarly, an increase in the width of the surround is followed by an increase in surround suppression (Paffen, van der Smagt, et al., 2005). Furthermore, it is in line with the aforementioned contrast matching tasks in which Xing and Heeger found that a narrow surround configuration produces less suppression than a wide surround configuration (Xing & Heeger, 2001). Combined with our results, these findings can be taken as evidence for a low-level summation effect.

### Exploratory findings

Although BR does not seem to be a feasible way to measure pseudoneglect, the design of the experiment made it possible to investigate nasal and temporal effects in the ambiguous condition in an exploratory manner. Our results demonstrate that when the surround was presented within the nasal visual half-field (presented to the temporal hemi-retina), predominance was stronger than when it was presented in the temporal visual half-field (presented to the nasal hemi-retina). We thus add to a large body of literature, demonstrating a strong advantage of the nasal half-fields in various other perceptual phenomena (Chen & He, 2003; Kaushall, 1975; Mustonen, Nuutinen, Vainio, & Häkkinen, 2018; Sahakian et al., 2022). Specifically, in one BR study investigating nasal and temporal differences, the stimulus presented within the nasal half-field (left visual field in the right eye) demonstrated extended dominance periods (Chen & He, 2003). Additionally, an earlier study used a simpler stimulus (two lines oriented CW or CCW) and found a nasal advantage in the form of prolonged dominance times (Kaushall, 1975). Furthermore, Sahakian and colleagues found that targets presented in the nasal visual half-filed broke through suppression quicker than targets in the temporal half-fields in breaking continuous flash suppression paradigms (Sahakian et al., 2022). We can contribute to this literature by noting that not only does the BR stimulus itself have a nasal advantage, but so does the spatially presented context of a central BR stimulus. Whether this enhanced processing is due to stronger processing in the visual system or an attentional benefit of the nasal half-field cannot be dissociated based on our data, but we could show that the enhanced processing of the surround-stimuli in the nasal half-field can lead to them exerting a stronger influence on central adjacent stimuli.

### Explaining the nonmatching boost effect in center-surround configurations

The results demonstrate a consistent and reliable nonmatching boost effect in center-surround configurations. This can be explained in the framework of figure-ground segregation, in which the center represents the figure and surround the ground. Thus, center-surround stimuli represent an entire image-based display, which is more easily interpretable if it can be divided into a figure and ground. In practice, the central rivalry target chosen is the one that stands out the most from the background, leading to a nonmatching boost effect. Figure-ground segregation is an essential principle of Gestalt psychology—whereas perceptual grouping determines the qualitative element of perception, figure-ground segregation determines the interpretation of those elements in terms of shape and relative location (Wagemans et al., 2012). This suggests that high-level effects play a prominent role in spatial context effects, which is further supported by the fact that the interocular condition was as strong as the intraocular condition.

## Conclusions

In our study, we investigated the influence of a surrounding annulus on BR and its interaction with the rivalry target. Regardless of ambiguity, contrast, or half-field, we observed a clear preference for the nonmatching grating. Ambiguity did not reduce the strength of this bias, unlike the low-contrast condition, which led to a reduction. This bias was approximately twice as strong when using a full annulus, indicating a low-level spatial summation effect. Notably, and surprisingly, there was no difference between the intraocular and the interocular condition, suggesting the involvement of higher cortical areas. Our findings suggest that the predominance of the nonmatching grating arises from figure-ground segregation. This effect is probably achieved by contrast-based lateral interactions between visual cortex neurons. It seems likely that those interactions are modulated and guided by top-down input from higher cortical areas.

## Supplementary Material

Supplement 1
