# Supplementary material for: Measuring the influence of contrast, ambiguity, and side of spatial context on perceptual dominance during binocular rivalry
Source: J Vis. 2025 Mar 21;25(3):6. doi: 10.1167/jov.25.3.6 (PMC11935559; doi:10.1167/jov.25.3.6)
Supplement: Supplement 1 [file jovi-25-3-6_s001.pdf]

Supplement

S.1. Methods – Stimuli Binocular Rivalry

Table S1.

Complete Overview of Experimental Stimuli (Full-Surround)

Surround Condition	One Eye	Rivalling Eye
ambiguous	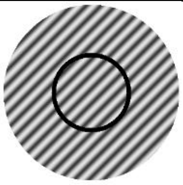	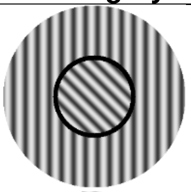
	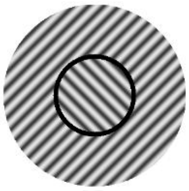	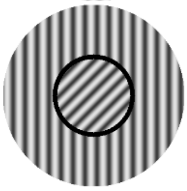
	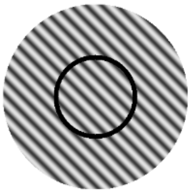	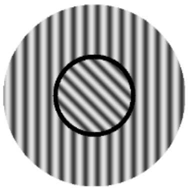
	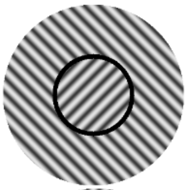	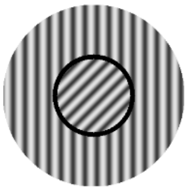
	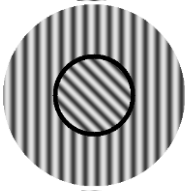	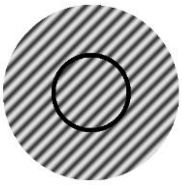
	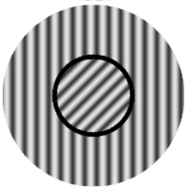	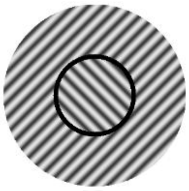
	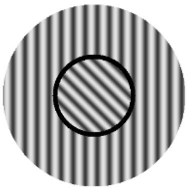	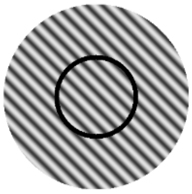
	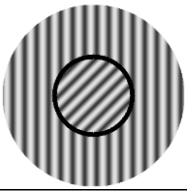	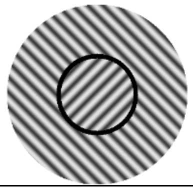

high contrast	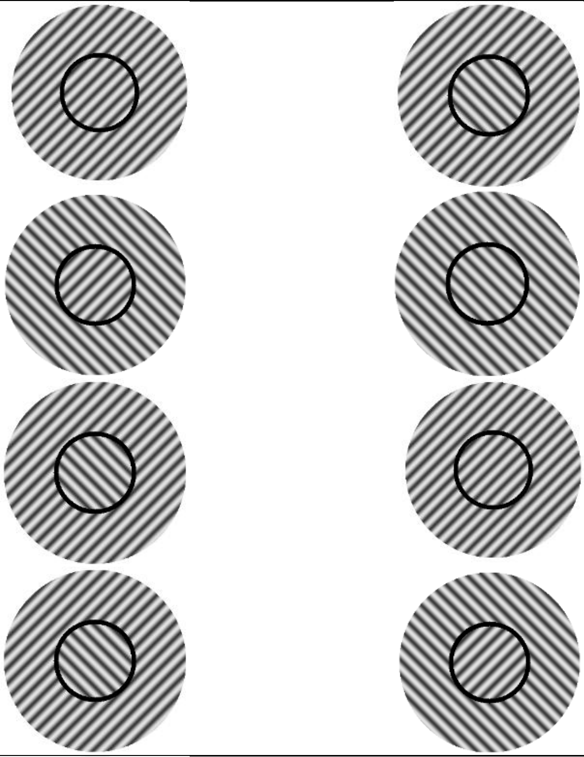
low contrast	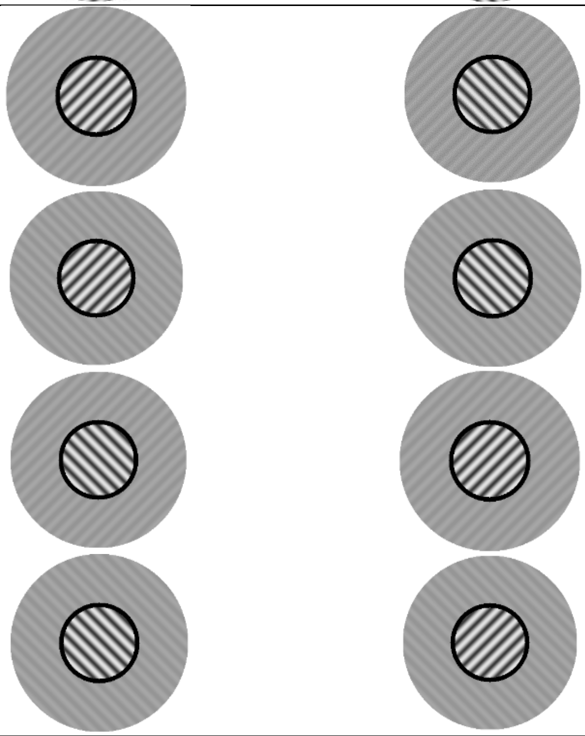

Table S2.

Overview of Experimental Stimuli with Half-Field Surrounds (Exemplified by Left Half-Field Surrounds)

Surround Condition	Nasal/Temporal	Left Eye	Right Eye
ambiguous	temporal (in the left eye)	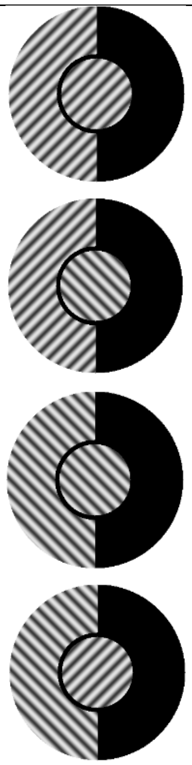	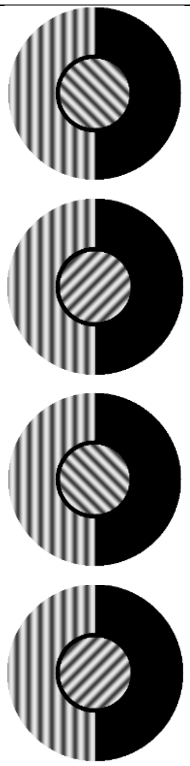
	nasal (in the right eye)	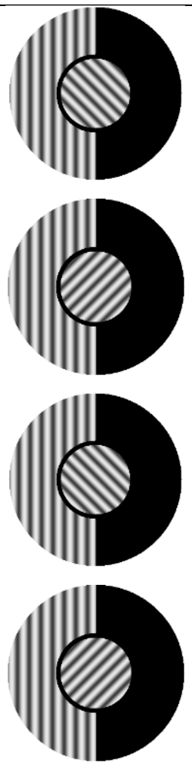	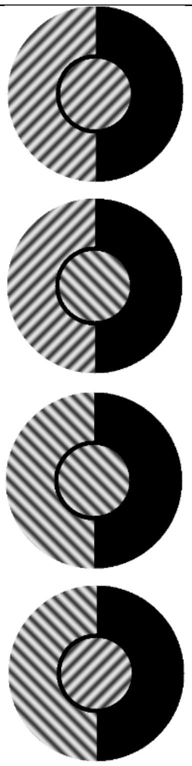

high contrast	both nasal and temporal	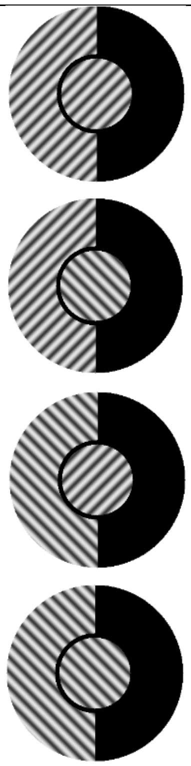	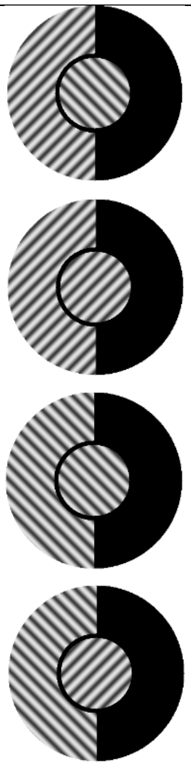
low contrast	both nasal and temporal	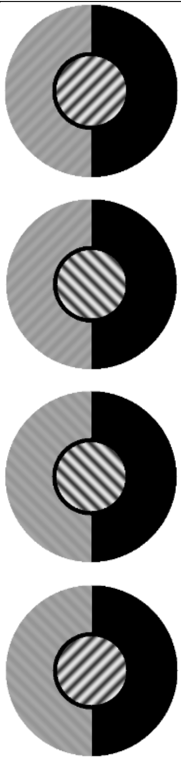	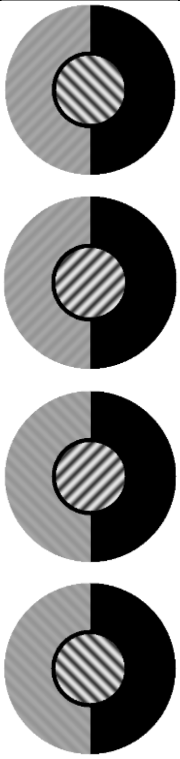

Note. Nasal/Temporal reflects the conditions used for our exploratory analysis. Only in the ambiguous condition can we have a nasal OR temporal condition. In the non-ambiguous conditions (high contrast, low contrast), the surround matching to one of the targets is always presented within the temporal AND

nasal half-field. For brevity, only conditions with a left half-field surround are shown; in the experiment, the half-field surround could appear equally often on the right side.

S.2. Methods – Procedure Binocular Rivalry

During the pilot phase, trials lasted 60 seconds each, and the experiment initially did not include low-contrast half-fields. Otherwise, the same trials were run in the main experiment but split by their surround. In one session, participants completed the half-field surround trials and, in the other the full surround trials. Changes were made to include the same conditions in full and half-fields to identify possible interactions. As the experimental time increased, we reduced the trial duration by 10 seconds. We cut off the pilot participants' responses at 50 seconds to have an equal trial duration for further calculations and make them comparable to the rest of the sample.

S.3. Ensuring Luminance Uniformity

We measured luminance for the average stimulus position. This position was estimated by measuring the position where stimuli fused the best for four different members of the lab. This measurement was done before we conducted the experiment, and six lookup tables with gamma-corrected luminance were created, one for each half-field and one for each rivalry target per side. This procedure ensured that all half-fields were equally bright during the trial and that any effect differing between the half-field conditions could be attributed to the experimental condition and was not confounded with possible luminance differences.

S.4. Results – Individual Participant Data

Figure S1.

Predominance on Individual Data: Effect of full surround for different contrast and ambiguity conditions (cf. Figure 1).

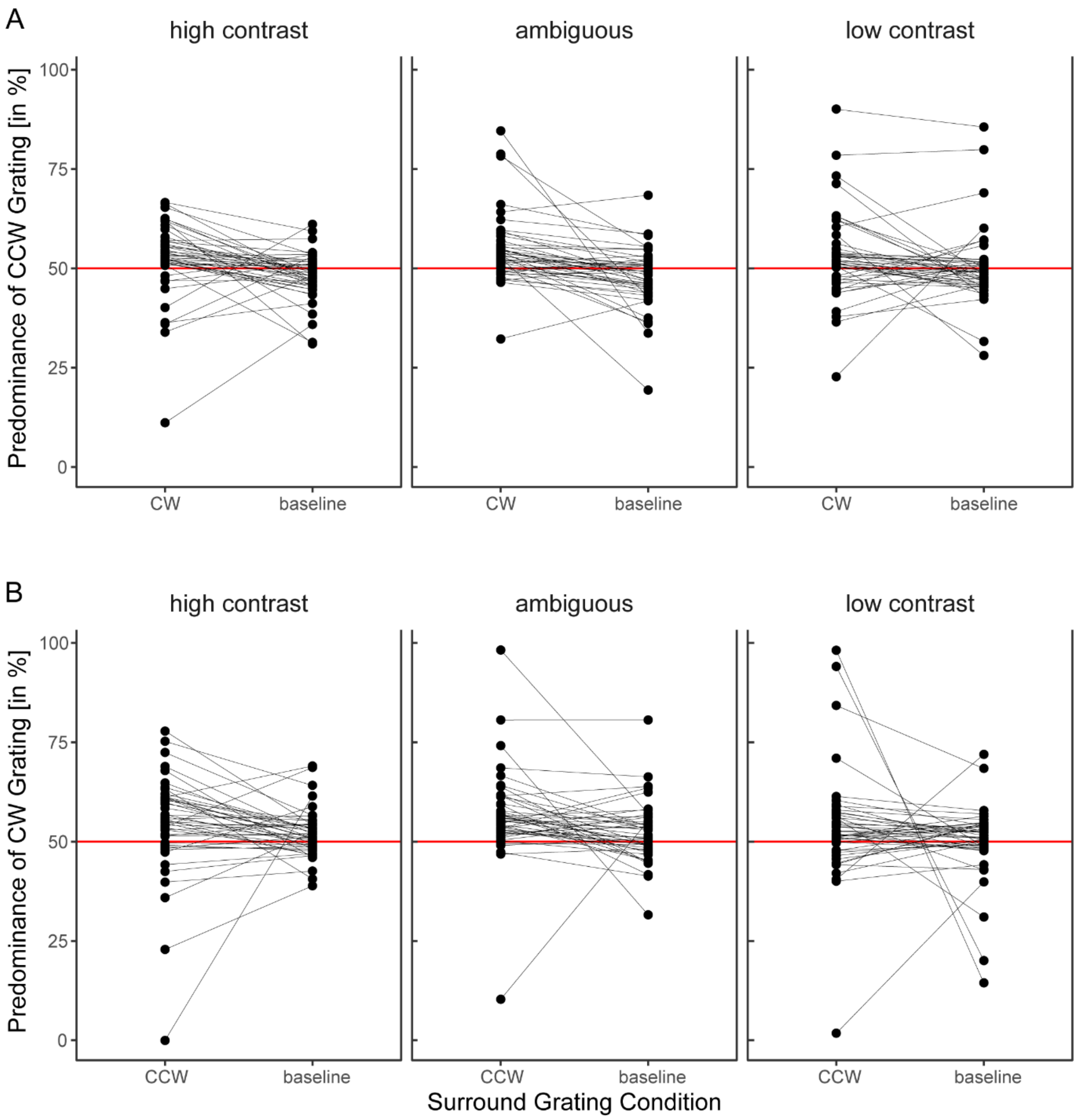

Note. Each point reflects the mean predominance of the non-matching target of a single participant. The lines connect data points of the same participant. In the significant conditions (high contrast, ambiguous), the majority show an effect in the same direction as the averages, i.e., CW > baseline and CCW > baseline.

Figure S2.

Mean Dominance Duration on Individual Data: Effect of full surround for different contrast and ambiguity conditions (cf. Figure 2).

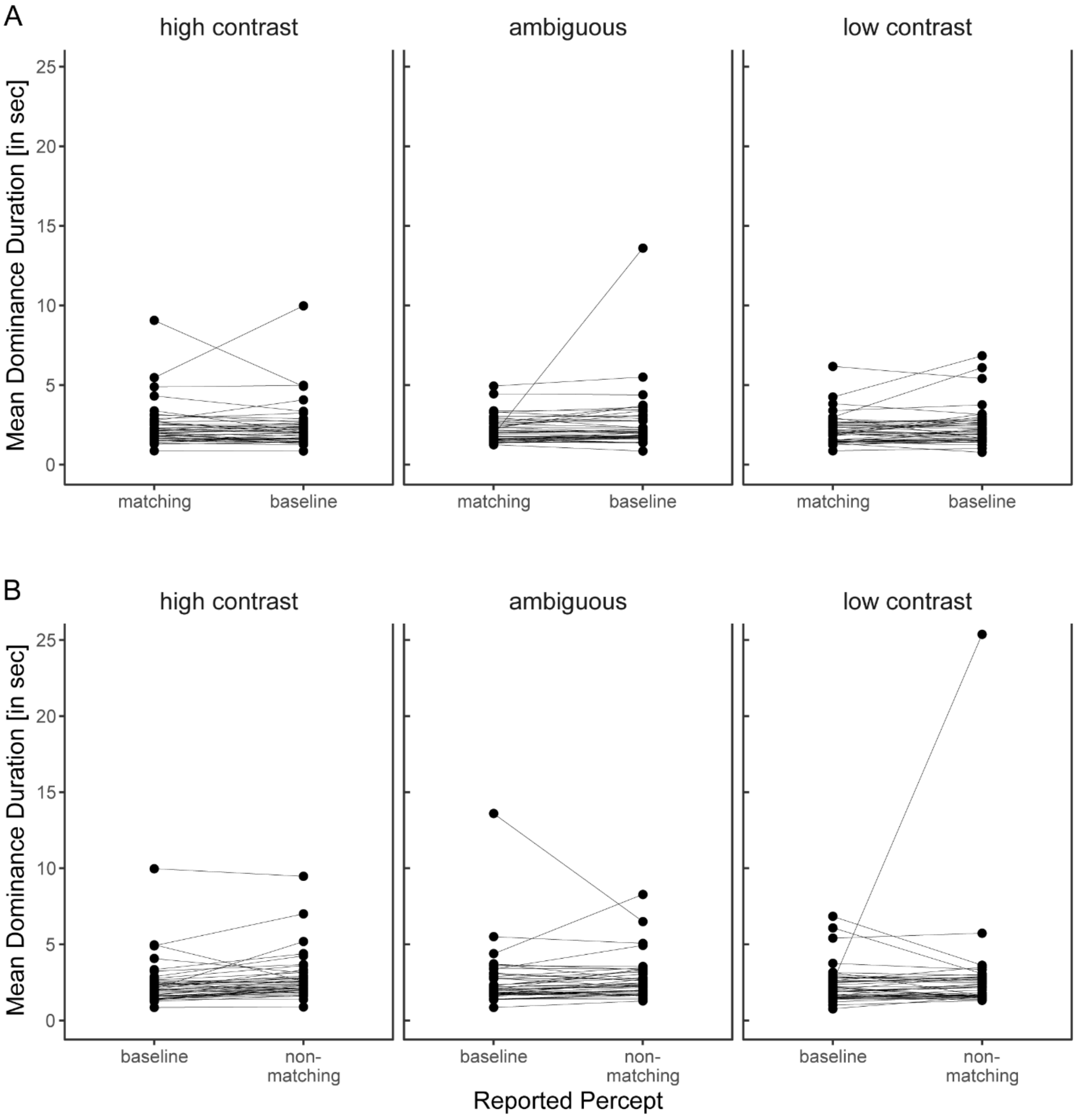

Note. Each point reflects the mean dominance duration of a single participant. The lines connect data points of the same participant. In the significant conditions (high contrast, ambiguous), the majority show an effect in the same direction as the averages, i.e., the mean dominance duration for non-matching > baseline.

Figure S3.

Predominance on Individual Data: Full vs. Half-Field Surround

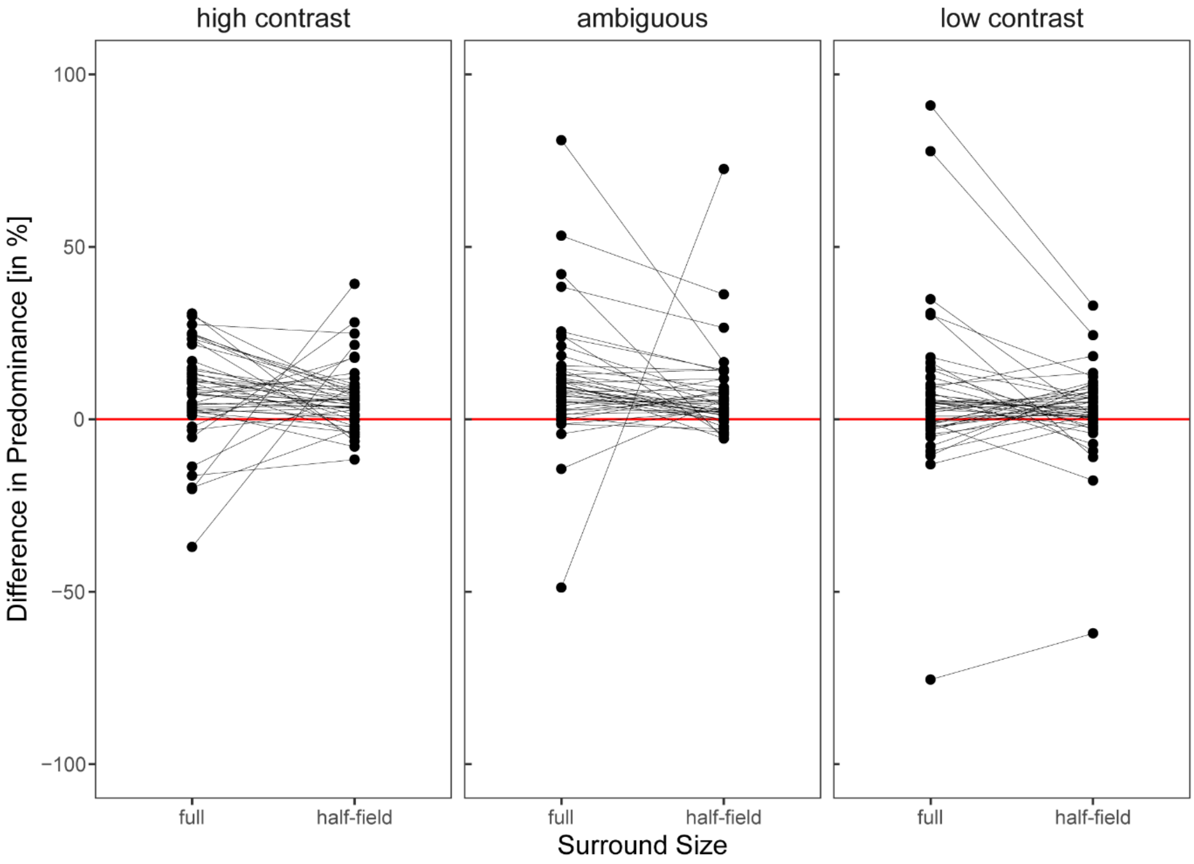

Note. Each point reflects the mean difference in predominance in the half-field and full-surround condition of a single participant. The lines connect data points of the same participant. In the significant conditions (high contrast, ambiguous), the majority show an effect in the same direction as the averages, i.e., full > half-field.
